# Pulmonary Hypertension in Chronic Lung Diseases: What Role Do Radiologists Play?

**DOI:** 10.3390/diagnostics13091607

**Published:** 2023-05-01

**Authors:** Adele Valentini, Paola Franchi, Giuseppe Cicchetti, Gaia Messana, Greta Chiffi, Cecilia Strappa, Lucio Calandriello, Annemilia del Ciello, Alessandra Farchione, Lorenzo Preda, Anna Rita Larici

**Affiliations:** 1 Division of Radiology, Fondazione IRCCS Policlinico San Matteo, 27100 Pavia, Italy; a.valentini@smatteo.pv.it (A.V.); lorenzo.preda@unipv.it (L.P.); 2 Department of Diagnostic Radiology, G. Mazzini Hospital, 64100 Teramo, Italy; paola.franchi@aslteramo.it; 3 Advanced Radiodiagnostic Center, Department of Diagnostic Imaging, Oncological Radiotherapy and Hematology, Fondazione Policlinico Universitario “A. Gemelli” IRCCS, 00168 Rome, Italy; lucio.calandriello@policlinicogemelli.it (L.C.); annemilia.delciello@policlinicogemelli.it (A.d.C.); alessandra.farchione@policlinicogemelli.it (A.F.); annarita.larici@unicatt.it (A.R.L.); 4 Diagnostic Imaging Unit, Department of Clinical, Surgical, Diagnostic, and Pediatric Sciences, University of Pavia, 27100 Pavia, Italy; gaia.messana@gmail.com; 5 Secton of Radiology, Department of Radiological and Hematological Sciences, Università Cattolica del Sacro Cuore, 00168 Rome, Italy; chiffi.greta@gmail.com (G.C.); strappacecilia@gmail.com (C.S.)

**Keywords:** pulmonary hypertension, chronic lung diseases, imaging, computed tomography

## Abstract

Pulmonary hypertension (PH) is a pathophysiological disorder, defined by a mean pulmonary arterial pressure (mPAP) > 20 mmHg at rest, as assessed by right heart catheterization (RHC). PH is not a specific disease, as it may be observed in multiple clinical conditions and may complicate a variety of thoracic diseases. Conditions associated with the risk of developing PH are categorized into five different groups, according to similar clinical presentations, pathological findings, hemodynamic characteristics, and treatment strategy. Most chronic lung diseases that may be complicated by PH belong to group 3 (interstitial lung diseases, chronic obstructive pulmonary disease, combined pulmonary fibrosis, and emphysema) and are associated with the lowest overall survival among all groups. However, some of the chronic pulmonary diseases may develop PH with unclear/multifactorial mechanisms and are included in group 5 PH (sarcoidosis, pulmonary Langerhans’ cell histiocytosis, and neurofibromatosis type 1). This paper focuses on PH associated with chronic lung diseases, in which radiological imaging—particularly computed tomography (CT)—plays a crucial role in diagnosis and classification. Radiologists should become familiar with the hemodynamical, physiological, and radiological aspects of PH and chronic lung diseases in patients at risk of developing PH, whose prognosis and treatment depend on the underlying disease.

## 1. Introduction

Pulmonary hypertension (PH) is a pathophysiological disorder, defined by a mean pulmonary arterial pressure (mPAP) > 20 mmHg at rest as assessed by right heart catheterization (RHC), according to the revised hemodynamic definition of the European Society of Cardiology (ESC)/European Respiratory Society (ERS) guidelines for the diagnosis and treatment of pulmonary hypertension, published in 2022 [1].

PH is not a specific disease, as it may be observed in multiple clinical conditions and may complicate a variety of cardiovascular and respiratory diseases.

Conditions associated with the risk of developing PH are categorized into five different groups, gathered according to analogous clinical presentation, pathological findings, hemodynamic features, and treatment strategy. In detail, group 1 PH includes all diseases characterized by pulmonary arterial hypertension (PAH), in which pathological changes primitively and predominantly affect distal pulmonary arteries; group 2 PH is associated with left heart diseases (LHD); group 3 PH includes lung diseases and/or hypoxic pathophysiology; group 4 PH is associated with pulmonary artery obstruction; and group 5 PH encompasses conditions with unclear and/or multifactorial mechanisms [1].

Based on hemodynamic aspects and according to the new ESC/ERS guidelines, PH is distinguished into: precapillary PH, characterized by a pulmonary vascular resistance (PVR) > 2 Wood units (WU) and normal pulmonary artery wedge pressure (PAWP ≤ 15 mmHg); post-capillary PH, characterized by low PVR (≤2 WU) and high PAWP (>15 mmHg); and combined pre- and post-capillary PH, characterized by PVR > 2 WU and PAWP > 15 mmHg, indicating pathological changes both in the venular and arteriolar sides [1].

Radiological imaging is involved in the diagnosis and follow-up of many diseases associated with PH, and many are imaging modalities that are helpful in the diagnostic work-up of PH [1,2]. Therefore, when monitoring conditions that can be complicated by PH, radiologists should evaluate possible indirect signs of PH, since its development is often associated with a significant increase in morbidity and mortality [3,4]. Furthermore, radiologists should be aware of their pivotal role in determining the potential causes of PH and in assisting clinicians toward a proper diagnosis. 

The correct clinical classification of PH is crucial for clinical management, prognosis determination, and therapeutic strategy choice, which significantly differ among the various PH groups. What is noteworthy is that the use of vasodilators is contraindicated in many clinical conditions outside group 1. 

This narrative review aims to highlight the advantages and limitations of the different imaging modalities employed in the diagnostic work-up of PH, to describe the radiological vascular/cardiac signs which allow for early detection of PH, and to deepen the knowledge of chronic lung diseases that can be complicated by the onset of PH, namely those included in group 3 and group 5 (Table 1), whose survival rates depends on the underlying disease.

## 2. Imaging Modalities

Although hemodynamic diagnosis is based on invasive RHC, multiple imaging modalities play important roles in the identification and classification of PH [5].

Multimodality imaging in PH diagnosis includes transthoracic echocardiography (TTE) and radionuclide ventilation/perfusion (V/Q) scanning. Among radiological modalities, chest X-ray (CXR), computed tomography (CT)/CT pulmonary angiography (CTPA), and cardiac magnetic resonance (CMR) are used. Pulmonary angiography has a role mostly in the planning of endovascular treatment (BPA: balloon pulmonary angioplasty; PEA: pulmonary endarterectomy) [2].

**Figure 1:**
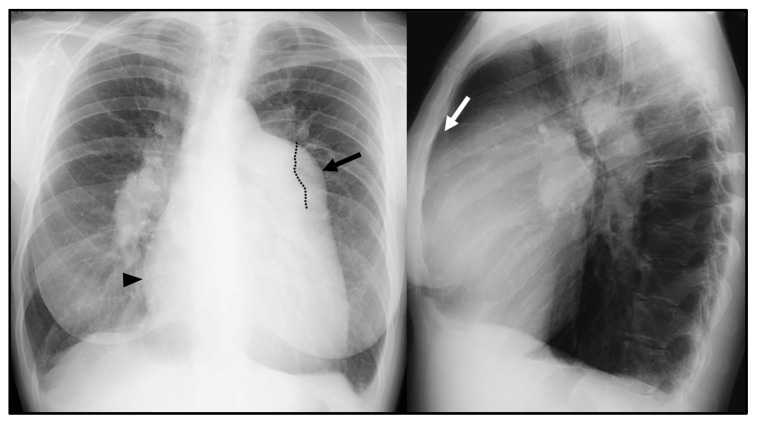
Chest X-ray (CXR) signs of pulmonary hypertension: enlarged central pulmonary arteries, with the prominence of the main pulmonary artery (MPA) (black arrow); the left pulmonary artery seen within the cardiac silhouette (dotted line); tapering of peripheral pulmonary arteries (so-called *pruning*); prominence of the right heart border, represented by the right atrium (black arrowhead); and filling of the retrosternal space in the lateral view due to right ventricle dilation (white arrow).

**CXR** is often the initial investigation in the evaluation of a patient with symptoms suggestive of PH. Even if frequently overlooked, CXR garners widespread use due to the relative ease of access, low cost [5,6], and potential capability of suggesting the presence of PH as well as the underlying cause [7]. The typical radiographic pattern of PH is characterized by the enlargement of central pulmonary arteries, associated with the tapering of the peripheral branches of pulmonary arteries, commonly referred to as *pruning* [7] (Figure 1). An enlarged right atrium (RA) and right ventricle (RV), commonly associated with advanced stage PH, can be evaluated on CXR as well [6]. The prominence of the right heart border (representing the RA) > 44 mm from the midline in the posteroanterior CXR and the filling of the retrosternal space in lateral film (representing RV dilation) are radiographic signs suggestive of right cardiac chamber enlargement [6]. A *boot-shaped* heart with an upward tilt of the cardiac apex can represent RV hypertrophy [8].

CXR showed a high sensitivity (97%) and specificity (99%) in the identification of PH [9,10]; nonetheless a normal CXR does not rule out diagnosis, particularly in patients with mild PH.

**CT** is commonly performed as part of the standard work-up for the diagnosis of PH. Due to its high spatial resolution, wide field-of-view, and capability of processing high-quality multi-planar reconstructions (MPRs) and maximum intensity projections (MIPs), CT allows the recognition of PH-induced anatomical changes in pulmonary vasculature up to the peripheral branches as well as the identification of possible etiologies of PH [2]. Particularly, high-resolution CT (HRCT) techniques facilitate the evaluation of lung parenchyma and the diagnosis of PH secondary to lung disease (group 3 and group 5 PH), while the use of contrast medium in CTPA is essential if chronic pulmonary embolism is suspected as a cause of PH (Group 4 PH) [11]. 

**Dual-energy CT (DECT)** has also been investigated to measure lung perfusion qualitatively and quantitatively [11]. DECT provides morphological information on the vasculature and functional information on perfusion by quantifying the iodine amount in the pulmonary vasculature, which can serve as a surrogate for pulmonary perfusion [12]. DECT is primarily used to replace V/Q scanning, which is not widely available, in the diagnosis of chronic thromboembolic PH (CTEPH), but it has also been investigated as a screening tool for PH brought on by any cause [11]. Nevertheless, the value of DECT in the clinical diagnostic work-up of patients with PH is still undetermined [1].

Radiation exposure represents the main limitation of CT imaging, even though new technologies enable a significant dose reduction compared to older-generation scanners [13]. 

**CMR** provides a global and reproducible assessment of the heart; hence, it is regarded as the reference standard for quantification of the RV volumes, mass, and function. As RV failure is the main cause of the symptoms and complications of PH, these parameters are crucial for prognosis [14]. 

**Figure 2:**
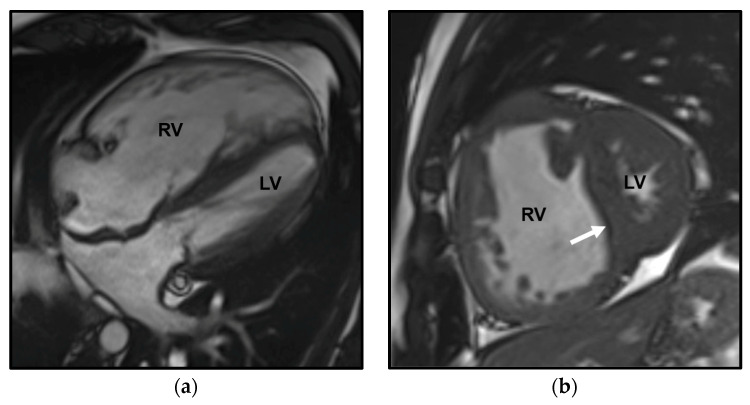

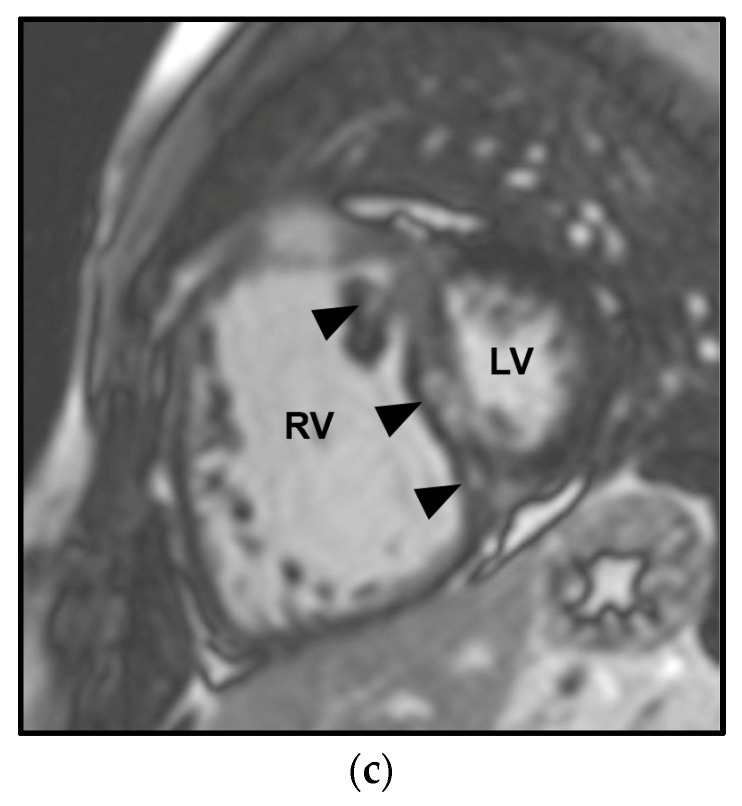
Cardiac magnetic resonance (CMR) in a patient affected by pulmonary arterial hypertension (PAH) in congenital heart disease with anomalous pulmonary venous return and intracardiac shunt. The Steady-state free precession (SSFP) in the four-chamber orientation in the diastolic phase shows marked dilation of the right ventricle (RV) and the compressed left ventricle (LV) (**a**). The SSFP in the short-axis orientation in the systolic phase depicts right ventricle dilation and leftward ventricular septal bowing (arrow) (**b**). The delayed enhancement CMR image (10 min post-gadolinium infusion) demonstrates mid-wall septal fibrosis and fibrosis at the anterior and posterior junctions between the septum and the RV free wall (arrowheads) (**c**).

**Figure 3:**
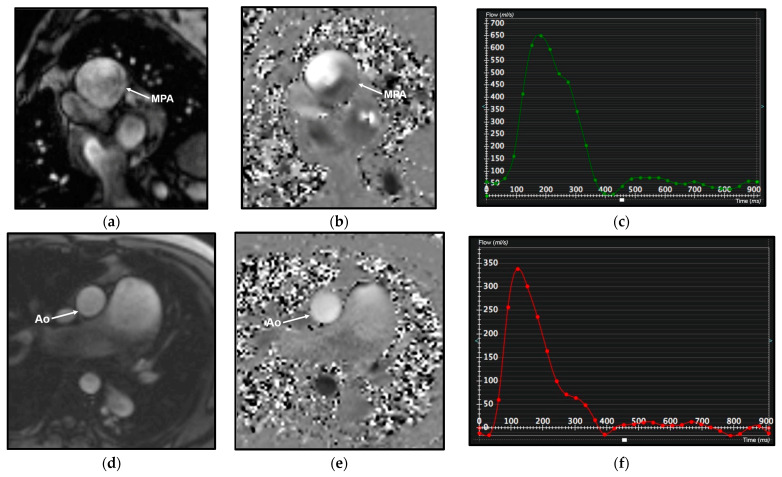
Cardiac magnetic resonance (CMR) phase contrast imaging in the same patient as in Figure 2. Magnitude (**a**,**d**) and velocity (**b**,**e**) images in phase contrast imaging through the main pulmonary artery (MPA) and aorta (Ao) for flow quantification; note the increase of the main pulmonary artery caliber, larger than the aortic one. Graphical representation of flow velocity in the MPA (**c**) and in the Ao (**f**) (*x*-axis: time in msec; *y*-axis: flow velocity in mL/s) demonstrates a markedly increased flow in the MPA compared to the Ao, with a calculated pulmonary blood flow (Qp) of 9.96 L/min and a systemic blood flow (Qs) of 3.45 L/min, with a Qp/Qs ratio of 2.8, indicative of left-to-right shunt.

CMR allows high-resolution time-resolved three-dimensional (3D) volumetric imaging of the right heart, quantification of blood flow, detection of congenital cardiac malformation/abnormalities—including shunts—and visualization of myocardial fibrosis and scarring [5] (Figure 2 and Figure 3). Moreover, the use of combined contrast angio-MR and pulmonary perfusion imaging, together with late-enhancement imaging of the myocardium, provides a comprehensive picture of both heart and pulmonary vasculature [1]. A limitation is the absence of an established method to estimate PAP [1]. 

In terms of follow-up, a systematic MR assessment of RV morphology and function permits the evaluation of PH under therapy and its response to treatment [15]. 

Finally, it is worth noting that the measurements performed on CT scans in patients with PH are equally applicable in CMR [5].

Apart from the cost, reduced availability, and the required technical expertise which precludes its use routinely, the main limitations of CMR remain the long acquisition time and inadequate evaluation of lung parenchyma [2]. 

This review focuses on the main role of CT in clinical practice for the assessment of patients with known or suspected PH.

## 3. CT indirect Signs of PH

**Figure 4:**
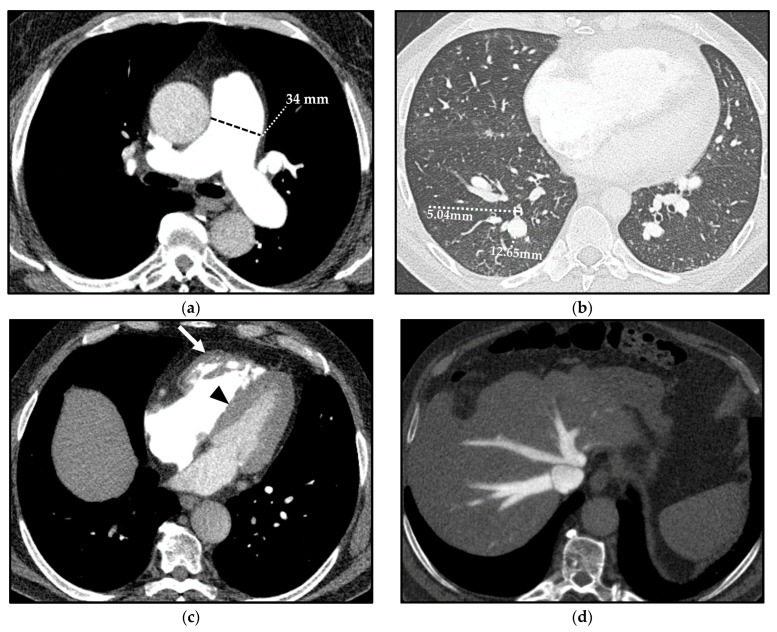
Computed tomography (CT) indirect signs of pulmonary hypertension (PH). Vascular CT signs: main pulmonary artery (MPA) dilation (34 mm) (**a**) and increased segmental artery-to-bronchus ratio (>1) (**b**). Cardiac CT signs: right ventricle (RV) hypertrophy, with free wall thickness > 4 mm and trabecular hypertrophy (arrow), and flattening of the ventricular septum (arrowhead) (**c**). Extensive reflux of contrast medium into the inferior vena cava and hepatic veins (**d**).

In patients with PH, the increase in PAP results in structural and hemodynamic changes that cause indirect vascular and cardiac CT signs, which should be recognized by radiologists (Figure 4). 

**The vascular CT signs** of PH reflect the underlying mural architecture of the vessels [16,17]. The multiple parallel elastic laminae that compose the wall of central pulmonary arteries (PAs) allow the caliber adaptation of main, lobar, and proximal segmental arteries to vascular bed resistance. On the other hand, a prevalent muscular component characterizes the distal segmental and subsegmental arteries, which are highly responsive to circulating factors and local stresses and are the most involved vessels in PH. Hypoxic vasoconstriction, vascular remodeling, and proliferative vaso-occlusive lesions usually occur at this distal level [16,17]. This is the reason why the earliest appreciable CT sign related to PH is the dilation of the proximal PAs and the sudden caliber decrease in peripheral ones [18].

A mean diameter of the main PA (MPA)—also known as the pulmonary trunk—(measured at its largest point within 3 cm of its bifurcation) ≥29 mm is predictive of PH with a sensitivity, specificity, and positive predictive value (PPV) of 87%, 89%, and 97%, respectively [19]; specificity increases up to 100% when the dilation of the MPA is matched with a segmental artery-to-bronchus ratio > 1 in 3 out of 5 pulmonary lobes on axial CT scans [19,20].

Moreover, the ratio between the diameter of the MPA and the diameter of the ascending aorta > 1 has been shown to have a sensitivity, a specificity, and a PPV of 70%, 92%, and 96%, respectively, for predicting PH in patients under 50 years of age [21]. Nonetheless, the negative predictive value (NPV) of this ratio is only 52%; thus, the absence of this parameter does not rule out PH [21]. 

In addition, no study has reported a significant correlation between the degree of dilation of the MPA and the severity of PH, and the usefulness of the measurement of MPA by CT is debated as it lacks standardization. The diagnostic efficiency of the MPA diameter measured on chest CT in identifying PH has provided inconsistent results also for the nonuniformity of patients’ selection [2]. Some authors have discredited the clinical relevance of the MPA diameter CT measurement in predicting PH in patients with interstitial lung disease (ILD) [22,23].

To limit this inconsistency, in the Fleischner Society position paper on PH, different cut-off values of the MPA dilation and PA diameter-ascending aorta diameter ratio (PA diameter-to-aorta) were proposed in populations at diverse classes of risk of developing PH. Indeed, the authors suggested a cut-off diameter of the MPA > 34 mm and a cut-off PA diameter-to-aorta > 1.1 in low-risk patients (less than 1%), >32 mm and >1.0 in intermediate-risk patients (1–10%), and >30 mm and >0.9 in high-risk patients (greater than 10%). It is worth noting that patients with chronic obstructive pulmonary disease (COPD), ILD, and systemic sclerosis (SSc) are included in the high-risk category [2].

A highly specific and easily recognizable sign of PH is the so-called *egg-and-banana* sign, whereby CT shows the superior aspect of the MPA at the same axial level as the aortic arch in severe PH [24].

**Cardiac CT signs** of PH depend on the exceeding adaptative mechanisms and subsequent failure of the RV. 

Prolonged and severe PH ultimately impacts on the RV, and this is manifested as hypertrophy followed by dilation of the ventricular cavity and straightening of the interventricular septum, causing an abnormal septal movement that finally injures the left ventricle (LV) function [25]. On CT, an RV free wall thickness > 4 mm is used to define “hypertrophy” [26]. A CT sign of RV dilation, on axial images, is characterized by an RV diameter greater than that of LV up to LV compression in severe cases [27]. Straightening or bowing of the interventricular septum yields a sensitivity and specificity of 86% and 91%, respectively, for PH identification, usually evident when the RV pressure is >30 mmHg [28]. 

Reflux of contrast into the inferior vena cava and hepatic veins on first-pass contrast-enhanced CT (e.g., CTPA) is a specific and indirect sign of right heart dysfunction [29].

An amount of fluid in the anterior pericardial recess between the MPA and the ascending aorta (resembling a *bikini bottom*) has been documented to be more frequent in patients with PH than in subjects with normal PAP, even though the pathogenesis is not fully understood [30] and this CT sign is not specific [24].

## 4. Group 3 PH

The distinction between group 3 PH associated with chronic lung diseases, namely ILD and COPD, and the other groups of PH classification is pivotal since group 3 is associated with the lowest overall survival among all groups, depending on the underlying disease. Indeed, the 3-year survival in PH associated with sleep-disordered breathing/alveolar hypoventilation (90%) is markedly better than PH associated with COPD (41%), which in turn is better than in the subgroup of ILD-PH (16%) [31].

### 4.1. ILD

ILDs that may be complicated by the development of PH are almost exclusively fibrotic ones, such as idiopathic pulmonary fibrosis (IPF), fibrosing hypersensitivity pneumonitis (FHP), combined pulmonary fibrosis and emphysema syndrome (CPFE), and connective-tissue-disease-related interstitial lung disease (CTD-ILD), which belong to group 3 PH. Of note, according to the latest ESC/ERS guidelines [1], lymphangioleiomyomatosis (LAM) associated with PH is also included in group 3, since a study performed on a large cohort of patients with LAM showed that PH was usually mild and associated with pulmonary function tests (PFTs) impairment, suggesting that the increased mPAP is strictly related to parenchymal abnormalities [32].

It is easy to assume that, as the lungs become fibrotic, the cross-sectional area of the pulmonary vascular bed is reduced, causing increased PVR and finally resulting in the development of PH. Nonetheless, the main cause of PH in ILD is chronic hypoxemia, which determines chronic pulmonary vasoconstriction and subsequent alterations of microcirculation [1,33,34]. Indeed, according to clinical studies, the presence of PH in fibrosing ILD significantly correlates with supplemental oxygen requirements [35]. 

To be regarded as the real cause of PH, ILD must show a typical radiological presentation coupled with certain PFT abnormalities, such as a reduction in forced vital capacity (FVC) of at least 70% and a diffusing capacity for carbon monoxide (DLCO) suggestive of a restrictive pattern, according to the “Proceedings of the 6th World Symposium on Pulmonary Hypertension” [36].

Therefore, radiologists should be able to properly recognize the HRCT signs of pulmonary fibrosis, such as reticular abnormalities associated with traction bronchiectasis, honeycombing, and volume loss, if present in patients with PH; these features must show an adequate extent of HRCT to justify the presence of PH, even though precise data on the minimal percentage of required lung involvement are still lacking [36].

Among fibrosing ILDs at risk of developing PH, most data are related to **IPF**. 

In IPF, the prevalence of PH varies according to disease severity. Indeed, it is lower in relatively early stages of IPF (8–14%), while it is common in advanced/end-stage disease (up to 86% at the time of lung transplantation) [31,33,36,37,38,39]. However, a correlation with the HRCT-based lung fibrosis score has not been demonstrated yet [23].

What is noteworthy is that the degree of PH in IPF is generally mild to moderate, with few subjects developing severe PH by the time of being listed for lung transplantation [35,37]. 

Moreover, in IPF patients, the presence of PH at diagnosis is associated with a high probability of subsequent development of acute exacerbation, resulting in a worse overall survival. In addition, mPAP has been shown to increase significantly over time because of acute exacerbations [38]. 

A severe form of PH may complicate **CPFE**, which is characterized by HRCT evidence of centrilobular and paraseptal emphysema in the upper lobes and fibrotic abnormalities in the lower lobes, with various patterns, from the more frequent usual interstitial pneumonia (UIP) to smoking-related interstitial fibrosis (SRIF) or a non-specific interstitial pneumonia (NSIP) pattern [39]. In CPFE, the prevalence of PH at diagnosis is 47%, which increases during follow-up to 55%. Patients with CPFE associated with PH have a poor prognosis, with only a 60% probability of survival after 1 year from the diagnosis of PH [40,41].

Nevertheless, in the case of severe PH, the ESC/ERS guidelines recommend excluding other possible causes of PH, such as LHD and CTEPH (groups 2 and 4, respectively), which may coexist with ILDs [1]. Furthermore, in patients with ILD and severe PH, a marked decrease in DLCO, disproportionate with respect to lung volumes, and a limited extent of fibrotic abnormalities on HRCT images, in the absence of CTEPH or LHD, should raise the possibility of a superimposed peripheral pulmonary vascular disease, falling into group 1 (pulmonary arterial hypertension, PAH) [36].

What is noteworthy is that even in patients who meet the criteria for the diagnosis of PAH, minimal interstitial lung alterations identified using PFTs and/or HRCT images have an important prognostic significance, because survival in these patients is worse than in patients with PAH without any parenchymal changes (1- and 5-yr survival of 95% and 70% in PAH without lung disease vs. 78% and 22% in PAH with mild lung disease, respectively) and the response to therapy with vasodilators is reduced [42].

Although PAH HRCT usually shows the absence of lung parenchymal abnormalities, it is possible to detect diffuse small centrilobular ground-glass nodules, which may represent cholesterol granulomas or recurrent pulmonary hemorrhage or plexogenic arterial lesions [25,34]; however, this finding does not influence patients’ prognosis [24,43,44,45,46]. According to some authors, ground-glass nodules are more commonly encountered in patients with idiopathic PAH (IPAH) who have been receiving long-term (years) vasodilator therapy [47].

Radiologists should recognize this finding in patients with PH as being generally associated with PAH rather than confusing it with even minimal interstitial disease or airways disease (e.g., non-fibrotic hypersensitivity pneumonitis), leading to a clinical misclassification and incorrect treatment strategy. In PAH cases, there are no radiological signs of pulmonary fibrosis and lung volumes are usually normal.

### 4.2. CTD/SSc-ILD

Compared to the general population, patients with **connective tissue diseases (CTDs)** have a higher risk of developing PAH (group 1), pulmonary veno-occlusive disease/pulmonary capillary hemangiomatosis (PVOD/PCH) (group 1), LHD-PH (group 2), ILD-PH (group 3), and CTEPH (group 4) mostly in the setting of antiphospholipid syndrome. Therefore, in daily practice, the precise phenotyping of PH in this patient category remains a challenge for clinicians.

**Systemic sclerosis (SSc)**, especially in its limited variant, is the main cause of PAH-CTD in Europe and in the United States (USA) [48,49,50], with a prevalence of pre-capillary PH of 5–19% [49,50], and therefore, it is included in group 1. However, precapillary PH in SSc may occur in association with ILD (group 3) and it is difficult to categorize these patients into a specific PH group according to a single pathological mechanism [51], since the presence of ILD could represent a confounding factor [36]. Nevertheless, in this patient category, it is important to understand and determine the main cause of PH to set a proper treatment strategy, bearing in mind that the development of PH is a negative prognostic factor [51,52]. 

On HRCT, SSc-ILD usually demonstrates an NSIP pattern or, less frequently, a UIP pattern. Goh et al. identified two risk categories in SSc-ILD patients, according to the extent of ILD abnormalities on HRCT, namely an extensive stage for ILD abnormalities > 20% and a limited stage for <20% [53]. Launay et al. performed a cluster analysis to define the different possible phenotypes encountered in patients with SSc and various degrees and combinations of both PH and ILD [54]. They demonstrated that in the case of limited-stage ILD on HRCT, patients’ prognosis is comparable to that of subjects with no lung involvement, thus depending solely on the severity of the hemodynamic profile and severity of PH (as in PAH); therefore, treatment may be aligned to that of PAH, based on vasodilators; on the contrary, in the extensive stage of SSc-ILD, the prognosis is always worse than in patients with limited stage, independently from the severity of PH (as in other forms of ILD-PH) [54]. For these reasons, according to the 2022 ESC/ERS guidelines, HRCT is recommended in SSc-PH patients to assess the presence of and quantify ILD changes [1].

Treatment with vasodilators is currently indicated in PAH but not in ILD-PH and in other diseases belonging to group 3 PH. In SSc patients with extensive ILD and PH, initiation of vasodilator therapy should be evaluated on a case-by-case basis because the response to therapy is inferior compared to patients with limited stage ILD or no evidence at all of ILD. Furthermore, it must be taken into account that vasodilator therapy in patients with extensive stages of ILD may cause clinical deterioration, possibly due to increased perfusion in non-ventilated fibrotic lung areas. Nevertheless, it should be pointed out that treatment with inhaled treprostinil, a prostacyclin analogue, has been recently approved in patients with PH-ILD (including SSc-ILD) [55].

Moreover, SSc can be associated with CPFE syndrome, and in these cases, the development of PH is related to a high mortality risk [56].

Sometimes, patients with SSc-PAH have features of venous/capillary involvement with a clinical and hemodynamic presentation such as PAH. Indeed, at histology, in association with typical findings of PAH, distinctive features of PVOD/PCH have been described, such as intimal fibrosis of septal and preseptal veins, fibrous broadening of the septa, patchy foci of interstitial capillary proliferation, and remodeled arterioles and venules [57]. Nonetheless, PAH and PVOD/PCH should be distinguished because of the worse prognosis of the latter, as well as for the possible occurrence of life-threatening pulmonary edema induced by PAH-targeted vasodilator therapy in PVOD/PCH patients. 

Three characteristic HRCT signs of PVOD/PCH include centrilobular ground-glass opacities, septal lines, and lymph node enlargement [58,59], findings also seen in pulmonary edema, but without central venous dilation and with normal appearance of the left heart chambers, in the presence of indirect signs of PH. The evidence of all three characteristic HRCT signs increases the likelihood of a correct diagnosis of PVOD/PCH in patients with SSc. However, also in the presence of only two of these signs, the probability of having PVOD/PCH in SSc patients is high and the risk of developing pulmonary edema due to PAH-targeted therapy is equally remarkable [57,60]. Furthermore, radiologists should be aware that the absence of these HRCT signs does not completely rule out the possibility of PVOD/PCH, especially in patients with suspected clinical diagnosis based on PFTs (DLCO < 50% of theoretical values) and arterial blood gas analysis (severe hypoxemia) [36,61]. 

**Figure 5:**
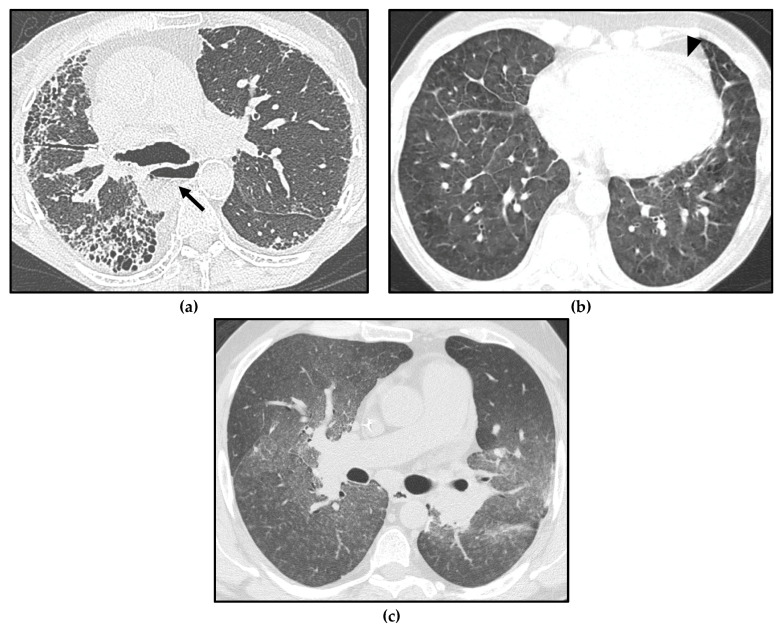
Different forms of pulmonary hypertension (PH) in systemic sclerosis (SSc) patients. Interstitial lung disease (ILD) showing a usual interstitial pneumonia (UIP) pattern, with asymmetric distribution of fibrotic abnormalities and honeycombing foci in the subpleural zones, especially in the right middle lobe and right lower lobe (**a**); note massive esophageal dilation, with the air–fluid level (arrow) and the enlargement of the main pulmonary artery (PA). Pulmonary veno-occlusive disease/ pulmonary capillary hemangiomatosis (PVOD/PCH), with smooth interlobular septal thickening and diffuse ground-glass opacities in the lung bases (**b**); note the mild pericardial effusion, which may be linked either to serositis or to PH (arrowhead). Pulmonary arterial hypertension (PAH) characterized by profuse bilateral tiny centrilobular ground-glass micronodules (**c**).

Isolated centrilobular ground-glass nodules can also be found in SSc-PAH [45], making the differential diagnosis with PVOD/PCH very challenging (Figure 5). Moreover, in SSc patients, pulmonary edema due to LHD or acute exacerbation of the underlying ILD, with radiological findings such as PVOD/PCH, can occur.

Therefore, a correct clinical–radiological correlation in a multidisciplinary context is required to distinguish between these conditions in SSc patients with PH and to avoid complications.

### 4.3. COPD

COPD is a common and treatable chronic lung disease, characterized by persistent respiratory symptoms (e.g., dyspnea, cough, and/or sputum production) and airflow limitation usually caused by exposure to noxious particles or gases, especially cigarette smoking [62]. A post-bronchodilator ratio of FEV1/FVC < 0.7 at spirometry is used to establish the presence of airflow limitation and confirms the diagnosis of COPD. To determine the severity of COPD, both functional tests (e.g., percent of predicted FEV1) and clinical data (e.g., symptoms scores and the number of exacerbations within the previous 12 months) are utilized [63,64].

The chronic airflow limitation in COPD is due to a combination of small airway disease and parenchymal destruction (emphysema), the relative contributions of which vary from patient to patient. HRCT enables to display of both lung damage due to emphysema with intraparenchymal or subpleural bullae and airway abnormalities, with the latter characterized by central bronchial wall thickening and distal airways changes, seen as solid centrilobular nodules sometimes with the appearance of a “tree-in-bud” pattern; moreover, expiratory scans can assess the presence of air trapping with a mosaic distribution [65]. 

PH in COPD (COPD-PH) is secondary to chronic hypoxia; nevertheless, a direct toxic effect of cigarette smoke seems to contribute to its development through pulmonary arterial remodeling with endothelial dysfunction and inflammation [66]. The prevalence of PH in COPD depends on the disease stage [36,67,68]. In end-stage COPD, PH prevalence reaches 90%, frequently with a mild to moderate severity, with only 1–5% of patients having mPAP > 35–40 mmHg at rest [69,70]. 

Although a strong association between the severity of PH and the extent of emphysema on HRCT images has not been demonstrated, neither visually nor by automatic evaluation of the parenchymal low attenuation area percentage (LAA%) [71,72,73], the extent of emphysema is often negatively correlated to alterations in lung microvasculature assessed by CT [74,75]. Furthermore, an analysis of anatomically matched airways showed that the mean bronchial wall thickness, evaluated with automatic software, is significantly higher in patients affected by COPD with PH than in those without PH [72]. 

**Figure 6:**
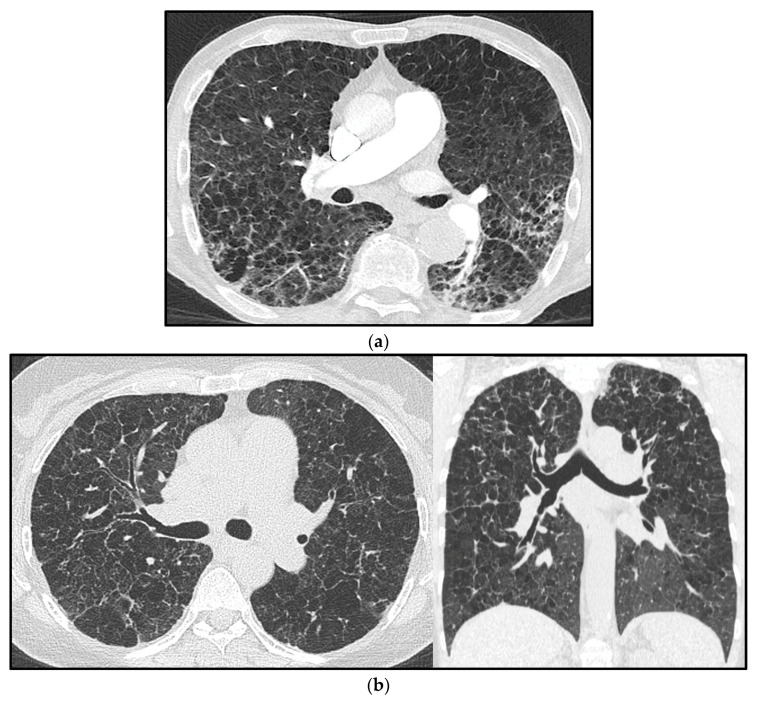
Pulmonary hypertension (PH) in a chronic obstructive pulmonary disease (COPD) patient with confluent centrilobular pulmonary emphysema (**a**). Multiple confluent bilateral irregular cysts in a patient affected by advanced pulmonary Langerhans’ cell histiocytosis (PLCH), complicated by PH (note the increased mean pulmonary artery diameter) (**b**); in advanced cases, the distribution of parenchymal abnormalities shows a rather diffuse longitudinal distribution, as seen in the coronal view, and the differential diagnosis with pulmonary emphysema is not straightforward.

From a practical point of view, to suspect the development of PH associated with chronic hypoxia, parenchymal abnormalities on HRCT should be evident (even in the absence of a validated threshold of disease extent) (Figure 6a), associated with reduced FEV1 (˂60% of the predicted value) and DLCO, consistent with obstructive/restrictive changes [36].

In patients with COPD and severe PH, DLCO is markedly reduced, indicating the probable involvement of vessels beyond the airways, as previously mentioned. In this respect, in a study conducted by Coste et al., the percentage of the total cross-sectional area of vessels normalized by lung area (%CSA) and the mean number of cross-sectioned vessels were greater in patients with COPD with severe PH compared to patients without severe PH. In addition, in patients with severe PH, a positive correlation was found between mPAP and the percentage of total CSA of vessels less than 5 mm^2^ normalized by lung area (%CSA < 5) [73].

## 5. Group 5 PH

This group includes disorders associated with PH, whose cause is either unclear or multifactorial; the cause of PH can be secondary to increased pre- and post-capillary pressure, as well as due to a direct effect on pulmonary vasculature [1]. Group 5 gathers hematological, systemic, metabolic disorders and miscellaneous diseases (e.g., chronic renal failure, pulmonary tumor thrombotic microangiopathy, and fibrosing mediastinitis).

Group 5 is the least investigated type of PH, despite constituting a prominent portion of the global burden of PH [76]. Diseases included in this group share poorly understood and composite pathophysiological mechanisms, such as hypoxic vasoconstriction, pulmonary vascular remodeling, thrombosis, fibrotic scarring and/or extrinsic compression of pulmonary vessels, pulmonary vasculitis, and cardiac failure, which may contribute to PH alone or in combination.

In this category of patients, a careful assessment to reach a diagnosis is needed, and treatment should be directed to the underlying condition [1]. Radiologists may play a crucial role in identifying imaging signs of PH or, in cases of known PH, suggesting the underlying disease and assisting clinicians with the correct treatment. Imaging is particularly useful in group 5 PH systemic disorders, which includes the spectrum of chronic lung diseases, such as sarcoidosis, pulmonary Langerhans’ cell histiocytosis (PLCH), and neurofibromatosis type 1 (NF-1). Lung parenchymal HRCT signs have been widely described in these disorders, some of which are highly suggestive, especially for the first two diseases, and radiologists should recognize them and, in addition, look for HRCT signs of PH in these diseases because this occurrence is associated with a worse prognosis.

In this section, we revised the HRCT appearance of the following lung diseases in association with PH.

**Sarcoidosis-associated pulmonary hypertension (SAPH)** is a quite common complication of sarcoidosis. The prevalence of PH in patients with sarcoidosis varies between 6% and 20% [77]. Even though the majority of SAPH patients show an advanced parenchymal disease, some subjects with limited parenchymal disease demonstrate disproportional elevated mPAP [78]. This means that apart from granulomata within the PAs and/or pulmonary veins and lung fibrosis, other mechanisms could determine PH in sarcoidosis, such as extrinsic compression of the pulmonary vessels by lymphadenopathies or fibrosing mediastinitis, vasospasm, vasculopathy, CTEPH, myocardial dysfunction, and portopulmonary hypertension [18,79,80]; indeed, pre- and post-capillary mechanisms may be both involved, sometimes in combination. Like other diseases complicated with PH, SAPH is associated with significant morbidity and increased mortality compared with sarcoidosis without PH [79,80].

Typical and atypical HRCT signs in sarcoidosis have been broadly depicted in the medical literature, with lymphadenopathies and pulmonary parenchymal changes representing the main findings of thoracic sarcoidosis. Enlarged mediastinal and hilar lymph nodes are the most common intrathoracic manifestations of sarcoidosis, typically with symmetric distribution and often containing fine eggshell-like calcifications [81,82].

The typical parenchymal appearance consists of small nodules (1–4 mm) with a perilymphatic distribution, usually bilateral and symmetrical, involving mainly the upper and middle lung zones [83]. Atypical HRCT manifestations of sarcoidosis are quite common and represent a challenge for the radiologist [82,83,84].

The use of PAH drugs in patients with SAPH has been investigated in small randomized controlled trials, which have suggested their efficacy in this context, but larger studies are needed to ensure their standard use [85]. In selected patients with active granulomatous disease, corticosteroids or immunosuppressive therapy may improve hemodynamics. When pulmonary vascular compression is suspected, pulmonary angiography may provide findings that require endovascular treatment. Anyhow, patients with SAPH have scarce long-term survival and, in selected severe cases, lung transplantation should be taken into consideration [1].

PH occurs in 92–100% of patients affected by advanced **PLCH** [86,87]. With respect to COPD and IPF, the incidence and severity of PH in patients with advanced PLCH are higher [18]. This is the reason why it has been postulated that other disease-related mechanisms (other than that of the ILD spectrum) may contribute to the development of PH in PLCH [88]. In particular, apart from fibrosis-mediated parenchymal and capillary destruction, endothelial dysfunction, hypoxic vasoconstriction, and the production of leukotrienes, additional mechanisms may occur with the increase in PAP, such as the elaboration of cytokines by the Langerhans’ cells of PLCH, which may exert vascular remodeling [89,90,91], direct invasion by immune cells (both Langerhans’ cells and the associated inflammatory milieu), which may influence vascular changes [87,89,92,93], and cystic formation, resulting in lung parenchymal destruction [92]. In 2018, Bois et al. demonstrated that in patients with PLCH, PAs within active lesions of PLCH have thicker intima and media than the PAs that are distant from active lesions; on the other hand, PAs distant from the active PLCH lesions also have significantly thicker intima and media than that of non-PLCH matched controls [94]. These results suggest vascular remodeling as an additional causative contributor to PH in PLCH [94]. So far, all postulated causative mechanisms of PH development in PLCH are pre-capillary.

In the appropriate clinical setting, radiologists can make a confident diagnosis of PLCH [95]. The pathological hallmark of PLCH is the accumulation of Langerhans’ cells and other inflammatory cells within the small airways, resulting in nodular inflammatory lesions. These nodules commonly cavitate and form cysts, representing enlarged airways. HRCT shows a predominantly nodular pattern in the early phases of the disease and a predominantly cystic pattern in the late phases; the cysts have been described as bizarre and usually spare the costophrenic recesses [95]. 

Advanced PLCH, when PH is usually more frequent, may have HRCT imaging features with large cysts and extensive lung parenchyma disruption, which overlap with emphysema and/or fibrosis as well as with LAM (Figure 6b). Therefore, in advanced cases, differential diagnosis among these entities may be challenging, and the typical radiological presentation of early PLCH (small nodules, sparing of posterior costophrenic recesses) make it difficult to recognize; it could be helpful to compare images with previous exams and to look for small cysts with bizarre morphology, peculiarly present in PLCH [18]. 

In 29 patients with PH associated with PLCH, PAH-targeted vasodilators improved hemodynamics without worsening oxygen levels [96]. In addition, in this case, lung transplantation represents a salvage therapeutic option for patients who have developed PH [97].

**PH associated with NF1 (PH-NF1**) is an uncommon but serious complication. Although the medical literature is poor, PH-NF1 seems to be characterized by female predominance, advanced age, and scarce long-term prognosis. PH-NF1 is associated with parenchymal lung changes in 2/3 of patients [91]; nevertheless, the evidence of a few cases with a severe form of PH but the absence of ILD raises the possibility of a specific pulmonary vascular disease [98]. The etiogenesis of PH-NF1 is poorly understood; histological samples of the pulmonary vasculature in PH-NF1 are limited to case reports, which report a pre-capillary dynamic mechanism that mainly involves PAs, with the development of intimal thickening and fibrosis, hyperplasia of pericytes and smooth muscle cells, and plexiform arteriopathy [99,100,101]. However, post-capillary etiology has also been postulated, due to the rare cardiac involvement in NF1 [98].

Thorax manifestations of NF1 are widely described and can be recognized by radiologists: chest wall neurofibromas (cutaneous and subcutaneous), kyphoscoliosis, ribbon deformity of the ribs, posterior vertebral scalloping, neurogenic tumors, meningoceles, bullous lung disease, and ILD [102]. 

Purely pulmonary manifestations of NF1 on HRCT are reported in 10–20% of adults, and include bullous, cystic, and/or interstitial parenchymal changes (such as diffuse ground-glass opacities, a mosaic pattern, and reticular opacities) [103,104]. ILD in NF1 is usually bilateral, symmetrical, and predominantly basal; it often occurs in association with thin-walled bullae, that exhibit an upper lung zone predominance [105]. On HRCT, neurogenic neoplasms of the posterior mediastinum are typically depicted in these patients [103]. Plexiform neurofibromas may appear as regular, circular, or oval masses in the paravertebral space or along the path of the vagus, phrenic, recurrent laryngeal, and intercostal nerves [106].

Despite the potential short-term benefit of PAH-targeted drugs, the prognosis remains poor, and lung transplantation should be considered in selected patients with severe disease [107].

## 6. Conclusions

In patients with chronic lung disease, the occurrence of PH is commonly associated with an increase in morbidity and mortality. A correct diagnosis is relevant for patients’ prognosis stratification as well as their treatment choices. Radiologists play a pivotal role on two fronts: the identification of signs of PH and, in patients with known PH, the recognition of eventual underlying chronic lung disease, thus guiding the most appropriate therapeutic strategies.

## Figures and Tables

**Table 1:** Chronic lung disease diseases associated with pulmonary hypertension (PH), clinical groups, and hemodynamic mechanisms.

Disease	Clinical Group	Hemodynamic
Fibrotic Interstitial Lung Disease *IPF* *fHP* *CPFE* *CTD/SSc-ILD*	Group 3: associated with chronic hypoxia	Pre-capillary PH
COPD	Group 3: associated with chronic hypoxia	Pre-capillary PH
Lymphangioleiomyomatosis	Group 3: associated with chronic hypoxia	Pre-capillary PH
Sarcoidosis	Group 5: multifactorial mechanisms	Pre-capillary PH Isolated post-capillary PH Combined pre–post-capillary PH
Pulmonary Langerhans’ cell histiocytosis	Group 5: related to pulmonary vascular dysfunction	Pre-capillary PH
Neurofibromatosis type 1	Group 5: multifactorial mechanisms with prevalent pulmonary vascular remodeling	Pre-capillary PH Combined pre–post-capillary PH

IPF: idiopathic pulmonary fibrosis; fHP: fibrotic hypersensitivity pneumonitis; CPFE: combined pulmonary fibrosis and emphysema; CTD: connective tissue disease; SSc-ILD: systemic sclerosis-interstitial lung disease; PH: pulmonary hypertension.

## Data Availability

Not applicable.
